# A novel echocardiographic right ventricular dysfunction score can identify hemodynamic severity profiles in left ventricular dysfunction

**DOI:** 10.1186/s12947-022-00290-5

**Published:** 2022-08-02

**Authors:** Odd Bech-Hanssen, Martin Fredholm, Marco Astengo, Sven-Erik Bartfay, Entela Bollano, Göran Dellgren, Kristjan Karason, Sven-Erik Ricksten

**Affiliations:** 1 Department of Clinical Physiology, Institute of Medicine at the Sahlgrenska Academy, Department of Molecular and Clinical Medicine, Sahlgrenska University Hospital, University of Gothenburg, Gothenburg, Sweden; 2 Institution of Medicine, The Sahlgrenska Academy at the University of Gothenburg, Gothenburg, Sweden; 3 Department of Anaesthesiology and Intensive Care Medicine, Gothenburg, Sweden; 4 Department of Cardiology, Gothenburg, Sweden; 5 Transplant Institute, Göteborg, Sweden; 6 Department of Cardiothoracic Surgery at, Sahlgrenska University Hospital, Gothenburg, Sweden

**Keywords:** Right ventricular dysfunction score, Hemodynamic profile, Left ventricular dysfunction, Echocardiography

## Abstract

**Purpose:**

Recognition of congestion and hypoperfusion in patients with chronic left ventricular dysfunction (LVD) has therapeutic and prognostic implications. In the present study we hypothesized that a multiparameter echocardiographic grading of right ventricular dysfunction (RVD) can facilitate the characterization of hemodynamic profiles.

**Methods:**

Consecutive patients (*n* = 105, age 53 ± 14 years, males 77%, LV ejection fraction 28 ± 11%) referred for heart transplant or heart failure work-up, with catheterization and echocardiography within 48 h, were reviewed retrospectively. Three hemodynamic profiles were defined: compensated LVD (cLVD, normal pulmonary capillary wedge pressure (PCWP < 15 mmHg) and normal mixed venous saturation (SvO_2_ ≥ 60%)); decompensated LVD (dLVD, with increased PCWP) and LV failure (LVF, increased PCWP and reduced SvO_2_). We established a 5-point RVD score including pulmonary hypertension, reduced tricuspid annular plane systolic excursion, RV dilatation, ≥ moderate tricuspid regurgitation and increased right atrial pressure.

**Results:**

The RVD score [median (IQR 25%;75%)] showed significant in-between the three groups differences with 1 (0;1), 1 (0.5;2) and 3.0 (2;3.5) in patients with cLVD, dLVD and LVF, respectively. The finding of RVD score ≥ 2 or ≥ 4 increased the likelihood of decompensation or LVF 5.2-fold and 6.7-fold, respectively. On the contrary, RVD score < 1 and < 2 reduced the likelihood 11.1-fold and 25-fold, respectively. The RVD score was more helpful than standard echocardiography regarding identification of hemodynamic profiles.

**Conclusions:**

In this proof of concept study an echocardiographic RVD score identified different hemodynamic severity profiles in patients with chronic LVD and reduced ejection fraction. Further studies are needed to validate its general applicability.

**Graphical abstract:**

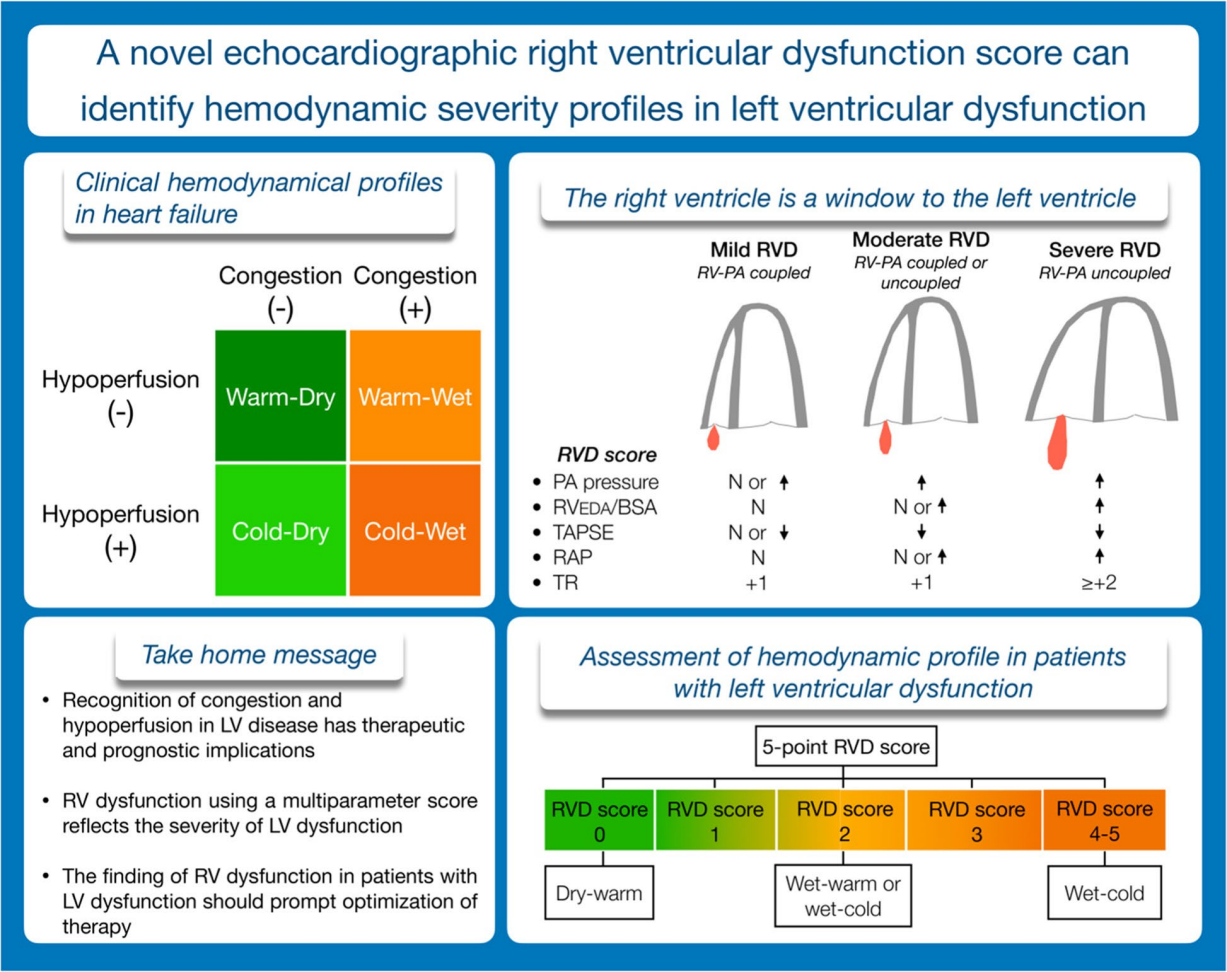

## Introduction

Echocardiography is a first line tool in the assessment of patients with suspicion of heart failure and important in the follow-up of patients with chronic left ventricular dysfunction (LVD) [1]. Elevated LV filling pressure and low cardiac output are invasive hallmarks of clinical overt LV failure (LVF). Over the last four decades echocardiographic assessment of elevated LV filling pressure has been a challenge and today a multiparameter approach is recommended [2, 3]. Patients with heart failure can be divided into different hemodynamic severity profiles based on the standard reference method, that is right heart catheterization. A patient with LVD is considered compensated (cLVD) if there are no signs of congestion (normal pulmonary capillary wedge pressure, PCWP) or systemic hypoperfusion (normal saturation of mixed venous blood, SvO_2_), decompensated (dLVD) if there are sign of congestion (increased PCWP) and in overt LVF if there are both signs of congestion and hypoperfusion. It is evident that grading the severity of LVD is important to guide therapy and risk-assessment. Still, the potential of echocardiography in this respect has gained little attention.

Increased right ventricle (RV) afterload due to increased LV filling pressures and secondary pulmonary hypertension is the most common cause of RV dysfunction (RVD) in patients with LVD [4]. The effect of LV backward failure on the pulmonary artery pressure, the RV afterload and RV-pulmonary artery coupling, is well recognized [5]. What is less well studied is how the different hemodynamic severity profiles in patients with chronic LVD effect the RV function, pulmonary circulation and the coupling between these. Since the extent of RVD is related to the degree of LV backward failure, it is likely that an echocardiographic assessment of RV function will mirror the degree of LVD. In the present study we hypothesized that using a novel RVD score [6], that is a lumped measure of severity of RVD, can facilitate the characterization of hemodynamic profiles in patients with chronic LVD.

## Methods

### Study population

We retrospectively identified 105 patients ≥ 18 years who were consecutively referred for heart transplant or heart failure work-up between July 2015 and July 2019, with right heart catheterization and echocardiography within 48 h. Exclusion criteria comprised 1) acute myocarditis; 2) myocardial infiltrative disease (e.g. amyloidosis and sarcoidosis); and 3) adult congenital heart disease. Among the total study population 72% performed a symptom-limited cardiopulmonary exercise test. Fasting blood samples were drawn in connection with the right heart catheterization. The study was approved by the Regional Ethics Review Board in Gothenburg (Dnr. 286–18) and conducted according to the Declaration of Helsinki.

### RVD score

The RVD score is empirical and the echocardiographic parameters included in the RVD score were selected because they are known to be common findings in patients with RVD or overt RV failure. The RVD score includes the following parameters and findings: 1) increased systolic pulmonary artery pressure (representing the common cause of RVD), 2) reduced tricuspid annular plane systolic excursion (TAPSE, representing longitudinal function), 3) increased RV diastolic area (a sign of RV-pulmonary artery uncoupling), 4) moderate or severe tricuspid regurgitation (a sign of RV-pulmonary artery uncoupling) and 5) reduced collapsibility of the inferior vena cava (a sign of increased right atrial pressure [5, 7, 8]. The first two parameters (pulmonary hypertension and reduced TAPSE) will be present in patients with any degree of RVD while the last three parameters (RV dilatation, moderate or severe tricuspid regurgitation and increased right atrial pressure), are more related to overt RV failure. Table 1 displays the cut-off values for the five parameters included in the RVD score, each parameter is assigned one point in the scoring system. The cut-off value for Doppler systolic pulmonary artery pressure ≥ 40 mmHg was chosen because this threshold is known to identify patients with a mean pulmonary artery pressure > 25 mmHg [9]. The grading of tricuspid regurgitation was performed taking into account the density and shape of the regurgitant jet, the color Doppler jet area and presence of systolic hepatic vein flow reversal [10]. Increased right atrial pressure (≥ 10 mmHg) was defined as collapsibility of inferior vena cava < 50%. For assessment of Doppler systolic pulmonary artery pressure, the graded assessment of right atrial pressure (3, 8, 10 and 15 mmHg) was done according to recent recommendations [11].

**Table 1:** Variables included in the RVD score and their cut-off values indicating RVD

	Parameter	Cut-off
RV afterload	Doppler SPAP (mmHg)	≥ 40 mmHg
RV size	RV_EDA_/BSA (cm^2^/m^2^)	> 12.6 (men), 11.5 (females)
RV longitudinal function	TAPSE (mm)	< 17
Tricuspid regurgitation	Multiparameter	≥ grade 2
RAP	IVC collapsibility (%)	< 50

*IVC* Inferior vena cava, *RAP* Right atrial pressure by echocardiography, *RV* Right ventricle, *RVD* Right ventricular dysfunction, *SPAP* Systolic pulmonary artery pressure, *RV*_*EDA*_*/BSA* Right ventricular end diastolic area indexed to body surface area, *TAPSE* Tricuspid annular plane systolic excursion

### Echocardiography

Standard assessment of LV and RV function was performed according to recent guidelines [11]. Increased LV filling pressures were assessed according to a previously proposed algorithm [3]. Cardiac output was calculated using standard pulsed-wave Doppler method and the lower limit for cardiac index was considered being 1.9 L/min/m^2^ for both genders [12]. The ratio RV end-diastolic area and RV long-axis was calculated and used as a RV sphericity index [13].

Speckle tracking was performed on the RV free wall by manual tracing. Only the middle and basal segments were used, and their mean defined as the RV free wall longitudinal strain. We omitted the apical segment due to often occurring (*n* = 24, 23%) problems with the visual assessment of tracking, mainly because the apical segment was only partly included in the sector.

### Hemodynamic measurements

A pulmonary artery catheter (7 F, Baxter Healthcare, Edwards Critical Care Division, Deerfield, Illinois, USA) was introduced using the Seldinger technique. The following variables were measured or derived: heart rate, right atrial pressure, RV end diastolic pressure, systolic pulmonary artery pressure, diastolic pulmonary artery pressure, mean pulmonary artery pressure, PCWP, cardiac output, pulmonary vascular resistance and SvO_2_. Cardiac output was determined by the thermodilution method as the mean of three to five consecutive measurements not varying by more than 10%. Stroke volume and cardiac output was indexed to body surface area yielding stroke volume index and cardiac index. Pulmonary vascular resistance was calculated as the difference between the mean pulmonary artery pressure and pulmonary capillary wedge pressure divided by cardiac output and expressed in Wood units. The pulmonary artery elastance was calculated as mean pulmonary artery pressure/stroke volume. The systolic RV elastance was calculated as systolic pulmonary artery pressure/end-systolic area [14].

### Statistical analysis

Continuous variables are expressed as the means ± standard deviation (SD), medians with interquartile ranges or as numbers with percentages when appropriate. The degree of the linear relationship was assessed by the Spearman correlation coefficient (R). To compare multiple groups, we used one-way ANOVA test when the distribution was normal and Kruskal–Wallis test when the distribution was skewed. In cases where the null-hypothesis was rejected (*P* value < 0.05 considered statistically significant), we continued with a post-hoc analysis of inter-group comparisons using the independent-sample t-test or Mann–Whitney test when appropriate. Using the Bonferroni correction for multiple testing, the null hypothesis was rejected if the *P* value was < 0.016. Receiver operator characteristic curve analysis were applied to assess the diagnostic ability to identify patients with 1) decompensation (PCWP ≥ 15 mmHg) and 2) overt LVF (PCWP ≥ 15 mmHg and SvO2 < 60%). The cut-off values for natriuretic peptide (NT-proBNP) and left ventricular ejection fraction were identified using the optimal Youlden index. The diagnostic performance was described using sensitivity, specificity, positive likelihood ratio and negative likelihood ratio. To evaluate the inter-observer variability of the RVD score the measurements and assessments were performed by two investigators (OBH, MA) on the same investigation (*n* = 20). The variability for continuous variables (TAPSE, RV diastolic area, Doppler systolic pulmonary artery pressure) was described by the coefficient of variation and expressed as the SD of differences divided by the mean value of two measurements. For categorical parameters (normal versus increased right atrial pressure, tricuspid regurgitation grade < 2 or ≥ 2, RVD score) we used kappa statistics. The statistical analysis was performed using SPSS for Macintosh, version 27 (IBM Corp, Armonk, N.Y., USA).

## Results

The mean ± SD age in the study group was 53 ± 14 years and 77% were males. Eighty-six patients were referred for heart transplant work-up. Eighty-eight patients had LV ejection fraction < 40%. Table 2 shows the patients’ clinical characteristics.

**Table 2:** Clinical characteristics

Etiology, %	
Dilated cardiomyopathy	65
Ischemic heart disease	21
Miscellaneous	14
Medical treatment, %	
Beta-blocker	94
Loop-diuretic	70
MCRA	49
ARB	37
ACE	37
ARNi	26
Anticoagulant	66
Devices, %	
ICD	43
CRT	34
Regular pacemaker	3
Htx work-up, %	
Listed for Htx	48
Not fulfil requirements	18
Considered ineligible	19

*ACE* Angiotensin-converting-enzyme inhibitor, *ARB* Angiotensin receptor II inhibitor, *ARNI* Angiotensin receptor-neprilysin inhibitor, *CRT* Cardiac resynchronization therapy, *ICD* Implantable cardioverter defibrillator, *Htx* Heart transplantation, *MCRA* Mineral corticoid receptor antagonist

Forty-two patients had cLVD with normal PCWP and SvO_2_ (dry-warm), 25 patients had dLVD with increased PCWP (wet-warm), 5 patients had normal PCWP and reduced SvO_2_ (dry-cold) and 33 patients had LVF with both increased PCWP and reduced SvO_2_ (wet-cold). Patients in the dry-cold group were due to the small number omitted from further comparison with the other three hemodynamic profiles.

### Relation between LV/RV function and hemodynamic severity profiles

**Fig. 1:**
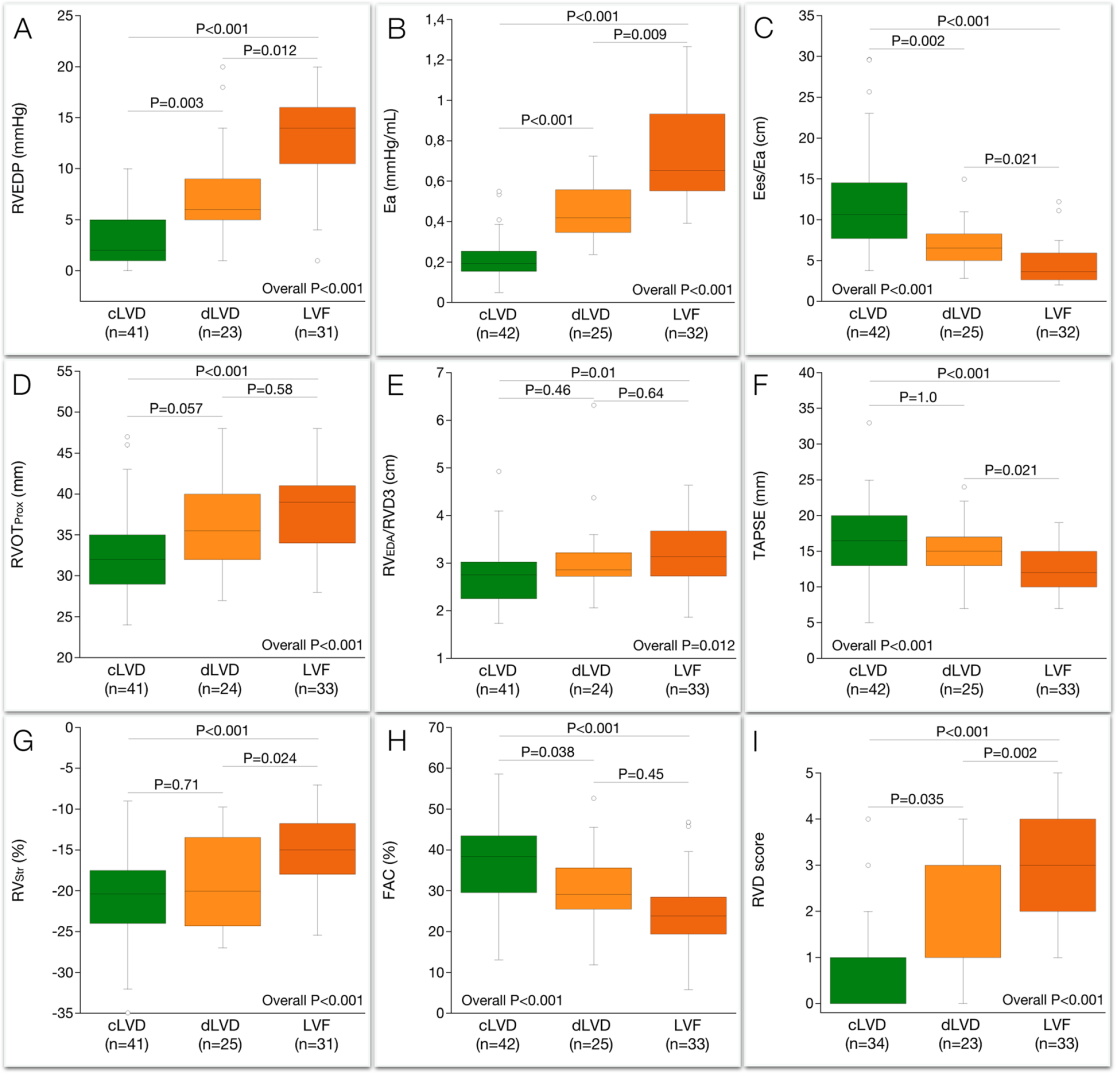
Box-plots showing the relationship between compensated left ventricular dysfunction (cLVD), decompensated left ventricular dysfunction (dLVD), overt left ventricular failure (LVF) and (**A**) the right ventricular end-diastolic pressure (RVEDP), (**B**) pulmonary artery elastance (Ea), (**C**) the ratio between right ventricular and pulmonary artery elastances (Ees/Ea), (**D**) the proximal diameter of the right ventricular outflow tract (RVOT_Prox_), (**E**) the ratio between the right ventricular diastolic area and the length (RV_EDA_/RVD3), (F) tricuspid annular plane systolic excursion (TAPSE), (**G**) right ventricular free-wall strain (RV_Str_), (**H**) fractional area change and (**I**) the right ventricular dysfunction (RVD) score. Significance values have been adjusted by the Bonferroni correction for multiple tests

Table 3 shows the clinical, laboratory, functional capacity, echocardiographic and right heart catheterization data in patients with cLVD, dLVD and overt LVF. The three groups with incremental impairment in LV function showed significant in-between groups differences in right atrial pressure and RV end-diastolic pressure (Table 3, Fig. 1A) with findings indicating a significantly larger proportion with RV failure in patients with LVF compared with the two other groups (Table 3). The RV afterload estimated as mean pulmonary artery pressure showed significant in-between differences for all three groups with signs of most pronounced pulmonary hypertension in patients with LVF. The total RV outflow impedance, estimated by the pulmonary artery elastance, was highest in patients with LVF and showed significant in-between differences for all three groups (Fig. 1B). Patients with LVF had significantly higher pulmonary vascular resistance (non-pulsatile afterload) compared with patients with cLVD. The RV-pulmonary artery coupling estimated as the ratio between RV and pulmonary artery elastances, showed significant in-between differences for all three groups with the most pronounced uncoupling in patients with LVF (Fig. 1C). As compared with patients with cLVD, patients with LVF had significantly larger and more spherical shaped RV (Fig. 1D-E). RV size and shape did not differ between patients with dLVD and LVF. Compared with dLVD, patients with LVF had significantly reduced longitudinal function (Fig. 1F-G). The longitudinal function did not differ between patients with cLVD and dLVD, but the global function did (Fig. 1H). The RVD score showed significant in-between differences for all three groups with the largest step-up between dLVD and LVF (Fig. 1I).

**Table 3:** Clinical, laboratory, functional capacity, echocardiographic and right heart catheterization data on patients with cLVD, dLVD and overt LVF

					Post hoc analysis^a^		
Variable	cLVD (*n* = 42)	dLVD (*n* = 25)	LVF (*n* = 33)	Overall p	cLVD vs dLVD	cLVD vs LVF	dLVD vs LVF
Clinical							
Age (years)	53 ± 13	54 ± 15	55 ± 14	0.70	-	-	-
Gender (male, %)	71	83	79	0.85	-	-	-
Dilated cardiomyopathy (%)	71	44	73	0.33	-	-	-
Ischemic heart disease (%)	19	48	18	**0.045**	0.08	1.0	0.07
Previous cardiac surgery (%)	5	16	9	0.35	-	-	-
Rales (%)	8	22	45	**0.045**	1.0	**0.036**	0.54
Edema (%)	21	18	35	0.55	-	-	-
Laboratory							
Hemoglobin (g/L)	140 ± 17	130 ± 13	137 ± 16	**0.047**	**0.04**	1.00	0.34
Creatinine (mmol/L)	95 (80; 129)	92 (84; 140)	125 (103; 143)	**0.012**	1.0	**0.02**	0.052
Bilirubin (µmol/L)	8.1 (6.0; 10.6)	12.0 (6.1; 18)	14 (12; 21.5)	** < 0.001**	0.46	** < 0.001**	0.058
NT-proBNP (ng/L)	1120 (571; 2155)	1980 (1280; 3690)	5040 (3340; 8395)	** < 0.001**	0.09	** < 0.001**	**0.009**
Functional capacity							
NYHA group 3–4 (%)	58	88	97	0.15	-	-	-
Ergo (W)	90 (76; 120)	90 (74; 109)	65 (53; 85)	**0.001**	1.0	**0.001**	**0.02**
Peak VO_2_ (mL/kg/min)	14 (12;18)	14 (11;17)	12 (9–13)	**0.002**	0.71	**0.002**	0.11
LV/LA dimension							
LV end-diastolic volume index (ml/m^2^)	85 (59; 112)	105 (92; 130)	109 (85; 141)	**0.009**	**0.04**	**0.02**	1.0
Left atrial volume index (ml/m^2^)	41 (33; 54)	62 (50; 68)	62 (51; 83)	** < 0.001**	**0.001**	** < 0.001**	1.0
LV function							
LV ejection fraction (%)	32 (25; 40)	25 (20; 32)	21 (17; 27)	** < 0.001**	0.09	** < 0.001**	0.36
LV global longitudinal strain (%)	-8.1 (-10.8; -6.1)	-6.5 (-9.0; -5.1)	-5.5 (-6.6; -2.6)	**0.001**	0.33	**0.001**	0.18
Doppler CI (L/min/ m^2^)	2.0 (1.8; 2.4)	1.8 (1.5; 2.1)	1.6 (1.1; 1.9)	**0.001**	0.27	** < 0.001**	0.23
Proportion with CI < 1.9 L/min/ m^2^ (%)	28	56	76	**0.015**	0.11	** < 0.001**	0.48
E/E’	12.1 (7.7; 17.8)	16.8 (13; 19.6)	16.3 (13.5; 21.3)	**0.03**	0.08	0.08	1.0
Increased left atrial pressure^b^ (%)	37	90	92	**0.028**	** < 0.001**	** < 0.001**	1.0
Mitral regurgitation ≥ moderate (%)	21	28	58	**0.024**	1.0	**0.005**	0.10
Right heart catheterization							
Heart rate	66 ± 11	73 ± 10	76 ± 12	**0.002**	0.71	**0.002**	0.16
MSAP (mmHg)	74 ± 14	73 ± 10	71 ± 8	0.96	-	-	-
Pulse pressure (mmHg)	50 ± 15	47 ± 18	40 ± 13	**0.013**	0.42	0.010	0.42
Right atrial pressure (mmHg)	2 (1; 4)	5 (3; 8)	11 (9; 14)	** < 0.001**	**0.008**	** < 0.001**	**0.002**
Right atrial pressure ≥ 10 mmHg (%)	2	12	67	** < 0.001**	0.42	** < 0.001**	** < 0.001**
SPAP (mmHg)	26 ± 7	42 ± 9	49 ± 9	** < 0.001**	** < 0.001**	** < 0.001**	0.20
MPAP (mmHg)	15 ± 5	28 ± 6	34 ± 6	** < 0.001**	** < 0.001**	** < 0.001**	0.10
PCWP (mmHg)	6 (4; 10)	18 (16; 21)	23 (20; 25)	** < 0.001**	** < 0.001**	** < 0.001**	0.08
Right atrial pressure/PCWP	0.31 (0.08; 0.50)	0.29 (0.15; 0.41)	0.48 (0.34; 0.65)	** < 0.001**	1.0	**0.002**	**0.001**
Stroke volume index (mL/m^2^)	39 ± 8	33 ± 8	25 ± 7	** < 0.001**	0.053	** < 0.001**	**0.002**
Cardiac index (L/min/m2)	2.7 ± 0.4	2.6 ± 0.8	2.1 ± 0.4	** < 0.001**	0.23	** < 0.001**	**0.016**
SaO_2_ (%)	95 ± 2	95 ± 2	94 ± 3	0.08	-	-	-
SvO_2_ (%)	70 ± 5	65 ± 4	52 ± 5	** < 0.001**	**0.02**	** < 0.001**	** < 0.001**
PVR (Wood units)	1.6 (1.2; 1.9)	1.9 (1.2; 2.4)	2.4 (1.8; 3.3)	** < 0.001**	0.35	** < 0.001**	0.08
RV/RA dimension							
RVD1 (mm)	42 ± 8	47 ± 10	50 ± 6	** < 0.001**	0.13	** < 0.001**	0.23
RV_EDA_/BSA (cm^2^/m^2^)	9.8 ± 2.5	11.4 ± 4.0	12.3 ± 2.8	**0.001**	0.28	** < 0.001**	0.23
RAAI (cm^2^/m^2^)	10.0 ± 3.5	12.3 ± 5.8	13.9 ± 3.6	** < 0.001**	0.20	** < 0.001**	0.07
RV function							
5-point RVD score	1 (0; 1)	1(1; 3)	3.0 (2; 4)	** < 0.001**	**0.03**	** < 0.001**	**0.002**
IVC collapsibility (%)	79 (55; 100)	64 (40; 78)	26 (17; 39)	** < 0.001**	0.21	** < 0.001**	**0.004**
S’ velocity (cm/s)	9 (8; 11)	8 (7; 10)	8 (6; 9)	0.12	-	-	**-**
Tricuspid regurgitation ≥ moderate (%)	7	16	45	**0.0015**	1.0	** < 0.001**	**0.03**

Bold represents significant *p* value

Values are mean ± standard deviation, median (IQR 25%;75%), or numbers and percent as appropriate. A *p*-value < 0.05 was considered significant

*BSA* Body surface area, *cLVD* Compensated left ventricular dysfunction, *dLVD* decompensated left ventricular dysfunction, *E/E’* ratio between early diastolic blood pool and annular velocity, *Ergo* Ergospirometry work load, *GLV*_*Str*_ Global left ventricular strain, *LVEDVI* Left ventricular end-diastolic volume indexed to body surface area, *LV* Left ventricle, *LVEF* Left ventricular ejection fraction, *LVF* Left ventricular failure, *MR* Mitral regurgitation, *MSAP* Mean systemic arterial pressure, *MPAP* Mean pulmonary artery pressure, *NYHA* New York Heart Association class, *PA* Pulmonary artery, *PCWP* Pulmonary capillary wedge pressure, *PVR* Pulmonary vascular resistance, *RAAI* Right atrial area index, *RV*_*EDA*_ Right ventricular area in diastole, *RVD1* Right ventricular inflow diameter, *RVD* Right ventricular dysfunction, *S’* peak systolic tissue velocity, *SvO*_*2*_ mixed venous oxygen saturation, *VO*_*2*_ maximal oxygen consumption

^a^Post-hoc analysis significance values have been adjusted by the Bonferroni correction for multiple tests

^b^Increased left atrial pressure using the proposed algorithm by the American Society of Echocardiography

### Detection of decompensation and overt LVF

**Fig. 2:**
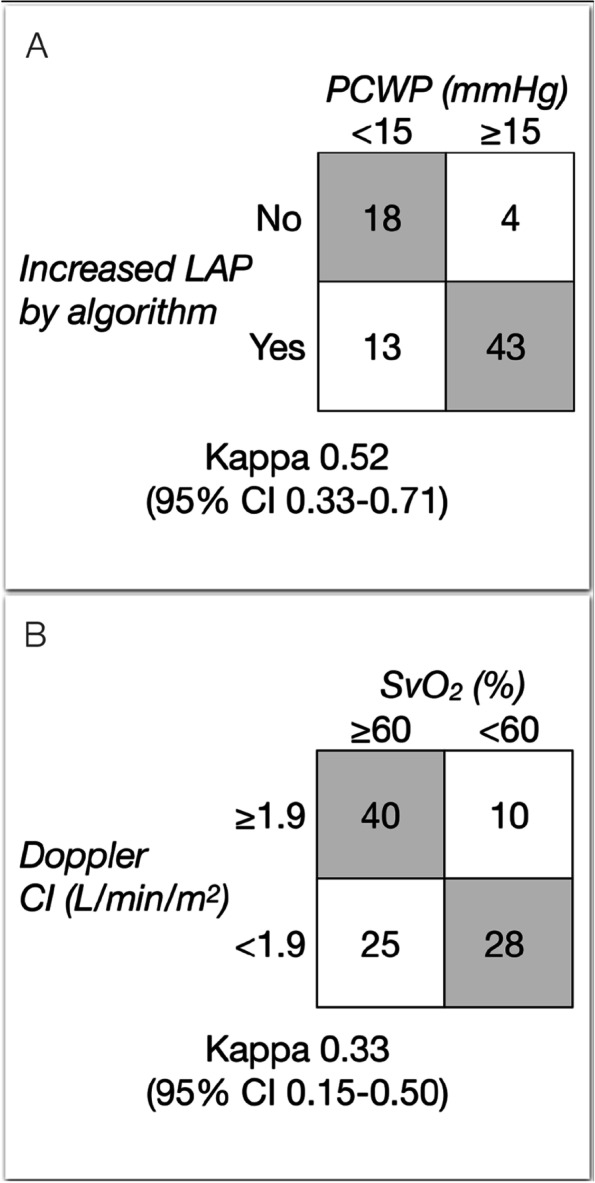
Crosstabs that shows the agreement between (**A**) increased pulmonary capillary wedge pressure (PCWP) at right heart catheterization and left atrial pressure (LAP) by echocardiography and (**B**) reduced SvO_2_ and Doppler cardiac index (CI)

Classification of the hemodynamic profile with standard echocardiography was performed in seventy-nine patients (75%). The classification was indeterminate due to missing Doppler cardiac index (*n* = 1, 1%), only one positive of two available parameters (*n* = 17, 16%) and less than two parameters (*n* = 8, 8%). The proportion of patients with correct echocardiographic assessment of hemodynamic profile was in cLVD 54%, in dLVD 44% and in LVF 73% (in-between groups *P* value 0.38). The agreement between the assessment of normal or increased left atrial pressure and invasive PCWP was moderate (kappa 0.52, 95% CI 0.33–0.72) with correct assessment in 78% and overestimation in 17% (Fig. 2A). The agreement between Doppler reduced CI, indicating hypoperfusion, and SvO_2_ was fair (kappa 0.33, 95% CI 0.15–0.50) with correct assessment in 66% and incorrect hypoperfusion in 24% of the patients (Fig. 2B).

Using standard echocardiography, the likelihood of decompensation (PCWP ≥ 15 mmHg), increased to a modest degree with positive likelihood ratio 2.8 (Table 4). The likelihood decreased to a large extent if LV filling pressure and cardiac index were considered normal. The likelihood of LVF increased to a moderate degree using standard echocardiography with positive likelihood ratio 2.9, and, on the contrary, the likelihood decreased to a moderate degree if both LV filling pressure and cardiac index were considered normal.

**Table 4:** Diagnostic performance of cut-off values to detect patients with decompensation and overt LVF

	Cut-off	Sensitivity (95% CI)	Specificity (95% CI)	PLR (95% CI)	NLR (95% CI)
Decompensated LVD (PCWP ≥ 15 mmHg)					
NT-proBNP	≥ 2000 ng/L	75 (60–83)	73 (58–84)	2.7 (1.7–4.8)	0.37 (0.23–0.59)
LVEF	< 27%	69 (56–79)	69 (54–81)	2.2 (1.4–3.6)	0.45 (0.29–0.69)
Standard echocardiography		91(80–97)	68 (49–82)	2.8 (1.7–4.9)	0.13 (0.05–0.33)
RVD score	≥ 1	96 (88–99)	41 (26–58)	1.6 (1.2–2.2)	0.09 (0.02–0.37)
RVD score	≥ 2	76 (64–86)	85 (70–94)	5.2 (2.3–11.8)	0.28 (0.17–0.46)
Overt LVF (PCWP ≥ 15 mmHg and SvO_2_ < 60%)					
NT-proBNP	≥ 3100 ng/L	82 (66–91)	77 (65–85)	3.5 (2.2–5.6)	0.24 (0.11–0.50)
LVEF	< 25%	61 (44–75)	69 (57–78)	1.9 (1.2–3.0)	0.57 (0.37–0.90)
Standard echocardiography		73 (54–86)	74 (60–85)	2.9 (1.7–4.9)	0.36 (0.19–0.70)
RVD score	≥ 2	97 (84–99)	72 (59–82)	3.5 (2.3–5.3)	0.04 (0.006–0.30)
RVD score	≥ 4	47 (31–64)	93 (83–97)	6.7 (2.4–18.4)	0.57 (0.41–0.80)

*CI* Confidence interval, *LVD* Left ventricular dysfunction, *LVF* Left ventricular failure, *NLR* Negative likelihood ratio, *PCWP* Pulmonary capillary wedge pressure, *PLR* Positive likelihood ratio, *RVF* Right ventricular failure, *RVD* Right ventricular dysfunction, *SvO*_*2*_ mixed venous saturation

**Fig. 3:**
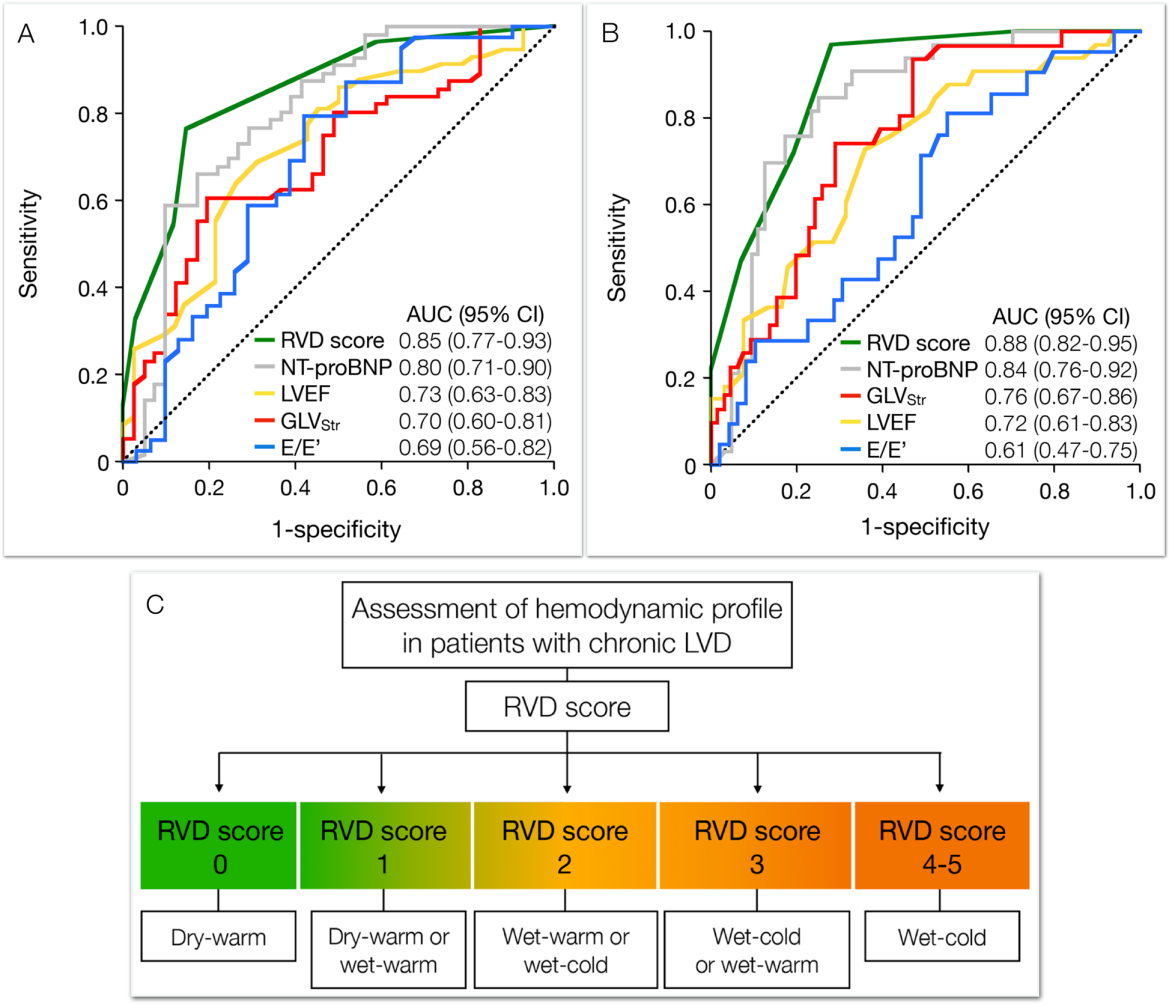
Receiver operator characteristic curves for detection of (**A**) decompensation and (**B**) overt left ventricular failure comparing the RVD score with NTproBNP, global longitudinal strain (GLV_Str_), left ventricular ejection fraction (LVEF), ratio between early diastolic mitral flow velocity and the tissue Doppler velocity (E/E’). (**C**) Diagnostic algorithm for assessment of hemodynamic profile in patients with chronic left ventricular dysfunction (LVD)

The ability of the RVD score, NT-proBNP, LV ejection fraction, global LV longitudinal strain and E/E’ to identify different hemodynamic profiles were tested using ROC analysis (Fig. 3A-B). As compared with LV ejection fraction, global LV longitudinal strain and E/E’, the area under the curve was largest for the RVD score and NT-proBNP (Fig. 3A). Using NT-proBNP levels, with a cut-off ≥ 2000 ng/L, the likelihood of decompensation increased to a modest degree, and decreased only slightly if the level did not reach the cut-off value (Table 4). With a cut-off value for the RVD score ≥ 2 the likelihood of decompensation increased considerably (rule-in threshold). Lowering the cut-off to RVD score ≥ 1 the likelihood of decompensation decreased substantially if the RVD score was below (RVD score 0 could be defined as a rule-out threshold). Similarly, as compared with LV ejection fraction, global LV longitudinal strain and E/E’, the RVD score and NT-proBNP had the largest area under the curve for detection of patients with LVF (Fig. 3B). Applying NT-proBNP level cut-off ≥ 3100 ng/L for detection of LVF increased the likelihood to a moderate degree if above or decreased the likelihood to a moderate degree if below the threshold (Table 4). Setting the RVD score cut-off level ≥ 4 the likelihood of LVF increased considerably (rule in threshold). Lowering the cut-off to RVD score ≥ 2 the likelihood of overt LVF decreased to a large extent if the RVD score was below (RVD score 0 or 1, rule out threshold).

### Inter-observer variability

The inter-observer variability for RV diastolic area, Doppler systolic pulmonary artery pressure and TAPSE was 8.8%, 8.6% and 22%, respectively. The agreement in echocardiographic assessment of right atrial pressure, tricuspid regurgitation and RVD score by kappa (95% CI) was 0.90 (0.71 to 1.0), 1.0 and 0.69 (0.47 to 0.92), respectively.

## Discussion

The main findings in the present study were as follows: firstly, the function of the RV-pulmonary artery unit is closely related to the LV hemodynamic profile; secondly, a standard echocardiographic assessment is less useful when it comes to identifying hemodynamic profiles; and thirdly, assessment of degree of RVD using a novel RVD score, can improve the assessment and differentiation of LVD beyond conventional echocardiography indices.

In patients with chronic heart failure and reduced LV ejection fraction, congestion and hypoperfusion at clinical examination are important findings with independent prognostic value beyond symptoms, biomarkers or chronic heart failure risk score [15, 16]. Bedside assessment of congestion is, on the other hand, hampered by reproducibility issues [17] and considered difficult and therefore often not thoroughly performed. Furthermore, increased LV filling pressure are known to precede symptoms and findings at clinical examination [18], and recognition of congestion with adjustment of therapy reduces the need for future hospitalization [19]. A non-invasive detection of fluid retention and increase in LV filling pressures has been a challenge throughout the history of Doppler echocardiography. In a recent meta-analysis the correlation between standard echocardiographic indices and invasive left ventricular filling pressures were only moderate [20]. A somewhat better approximation could be achieved by applying a multiparameter approach [3], but the methodology needs further validation [2, 20–22].

**Fig. 4:**
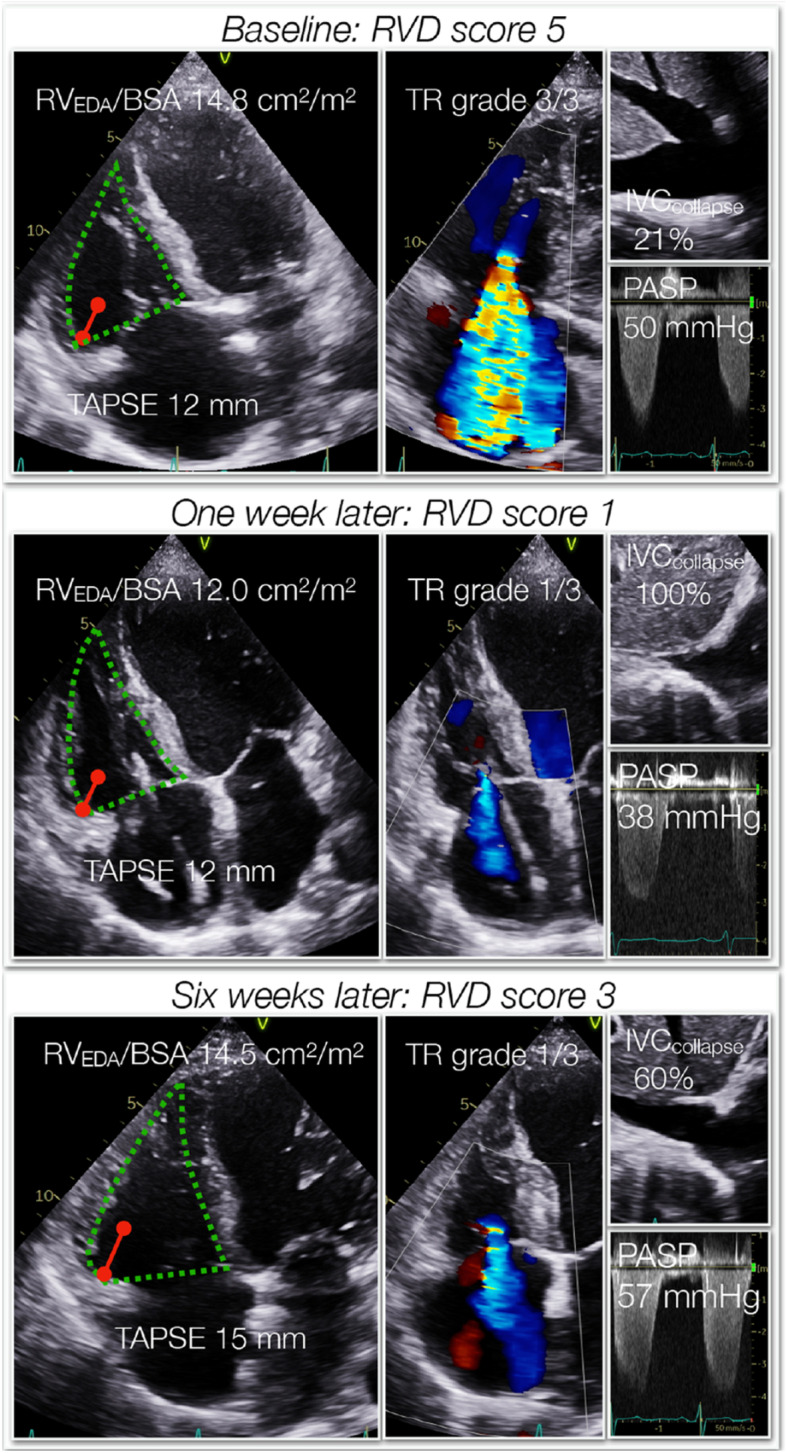
The dynamic nature of RVD illustrated by a patient with ischemic cardiomyopathy. At baseline (upper panel) the patient had biventricular failure with at catheterization PCWP 30 mmHg, RVEDP 17 mmHg, SvO_2_ 45% and NT-proBNP 5000 ng/L. The RVD score was 5 with moderate pulmonary hypertension, reduced TAPSE, RV dilatation, increased central venous pressure and severe tricuspid regurgitation. One week later (middle panel) after treatment with levosimendan and intravenous furosemide, NT-proBNP was 1080 ng/L and the RVD score was 1 due to reduced TAPSE. Six weeks later (lower panel), the patient was in NYHA class IIIb, NT-proBNP was 2370 and the RVD score 3. The hemodynamic profile from the RVD score indicated at baseline that the patient was wet-cold, one week later dry-warm or wet-warm and after 6 weeks wet-cold or wet-warm

In the present study, we sought for other indices that could identify different hemodynamic profiles. We hypothesized that a score system consisting of multiple parameters related to RVD could improve the detection and differentiation of hemodynamic profiles [6]. Our results show that a stepwise impairment in LV function is associated with a corresponding augmentation of the RV afterload, higher RV filling pressure, RV dilatation, reduced longitudinal RV function and more frequent moderate or severe tricuspid regurgitation. Using specific rule-in and rule-out thresholds the RVD score was more helpful than standard echocardiography and NT-proBNP regarding identification of hemodynamic profile. It is important to note that patients with RVD scores 1–3 are indeterminate regarding specific hemodynamic profiles, but the likelihood of more deranged LV function will increase from RVD score 1 (most likely dry-warm) to RVD score 3 (most likely wet-cold) as illustrated in (Fig. 3C). The relation between RVD score and hemodynamic profile indicate that a stepwise increase in the RVD score in a patient raises the suspicion of LV hemodynamic deterioration (Fig. 4). The RVD score is probably highly dynamic and will reflect changes in LV hemodynamic status as such variations will cause alternations in RV loading conditions. Further studies are required to confirm to what extent the RVD score can be used to monitor individual fluctuations in LV hemodynamic profiles.

During the last two decades, the RV function in patients with LVD has gained increased attention since RVD has been shown to carry prognostic information beyond traditional markers of LVD [23–25]. These studies have in common that they have included patients with systolic heart failure and a limited range of LV ejection fraction. Therefore, it is not a surprise that the different RV function parameters that were investigated had stronger predictive ability in multivariate models than LV ejection fraction. The different studies all conclude that poor prognosis is mainly related to the degree of RVD. The results of our study suggest a clinically important alternative interpretation. The RVD appears to be of prognostic importance since it reflects the severity of LVD. The individual longitudinal (TAPSE, RV strain) and global (fractional area change) parameters differed significantly between the three hemodynamic profiles, however, only the RVD score showed significant in-between differences for the three groups. Importantly, given the causal link between LV and RV dysfunction, a marked improvement in RV function can be observed following improvement of LV function after optimization of heart failure treatment (Fig. 4) [26]. Therefore, observing RVD, preferably using a multiparameter approach, in a patient with LVD should prompt optimization of therapy. That being said, it is important to realize when RVD is caused by intrinsic muscular disease rather than hemodynamic overload secondary to LVD. This is often the case in infiltrative myocardial diseases or when end stage heart failure has caused permanent RV damage with myocardial fibrosis [27]. Certain clinical and laboratory findings may support the diagnosis of end stage RV failure. Severe tricuspid regurgitation and RV dilatation without increased LV filling pressures or pulmonary hypertension are examples of echocardiographic signs supportive of genuine RVD.

## Limitations

The most important limitations to discuss are related to the general applicability of our findings. Firstly, the study was retrospective and dominated by patients with advanced heart failure. To what extent our results can be extrapolated to patients with chronic but less symptomatic LVD is of crucial importance. Secondly, the study patients had predominantly heart failure with reduced LV ejection fraction. RVD in patients with heart failure and preserved ejection fraction is known to be a major predictor of the clinical outcome [28]. Therefore, a replication phase is required including external validation of our results and involving evaluation of the echocardiographic RVD score in other heart failure populations.

In the present study we hypothesized that degree of RVD is coupled to the degree of LVD. This coupling will be less tight if the underlying disease process engage the RV myocardium. We excluded patients with myocarditis and infiltration disease, still, there is a possibility that the RVD observed in our patients to a degree that we cannot estimate, is caused by the underlying disease process involving both ventricles.

Today RV free-wall strain is of many considered a better descriptor of RV function than TAPSE. Still, in the present study we chose TAPSE for mainly two reasons. Firstly, due to often occurring problems related to inclusion of the apical segment and the assessment of the quality of the tracking, the proportion of patients with 3-segment free-wall strain will be less than with TAPSE, that can be achieved in almost 100%. Secondly, free-wall strain is known to be vendor-dependent and, strictly, any proposed cut-off indicating RV dysfunction can only be used if the same vendor is used.

In the present study assessment of RV size was based on 2D area and linear dimensions. It is well known that due to the complex RV anatomy, dilatation can pass unnoticed by echocardiography. Therefore, it is conceivable that future use of 3D based echocardiography can improve the detection of RV dilatation and allow assessment of RV ejection fraction [29].

## Conclusions

In the present study we found that a 5-point echocardiographic multiparameter RVD score identified patients with different LV hemodynamic severity profiles. This method outperformed the assessment offered by standard echocardiographic measurements. The RVD score, which is based on the pathophysiology of RV decompensation, is easily obtained from a standard echocardiographic examination. The standard echocardiographic assessment of LV filling pressure and cardiac out-put are hampered by a substantial number of false positives. In that context, in clinical practice, assessing the RV function using an easily obtainable multiparameter RVD score can give additional support for a patient being compensated (RVD score 0) and with increasing score increasing likelihood of decompensation or overt LVF.

## Data Availability

All raw data are readily available per request.
